# Prevalence and molecular characterization of multidrug resistant *Campylobacter* isolated from animals and humans as a one health approach

**DOI:** 10.1038/s41598-025-13120-1

**Published:** 2025-08-18

**Authors:** Mohamed Kholeif, Mohamed G. Sayed, Ahmed Fotouh, Doaa. A. Abd-Allah, Rania M. Ewida, Ahmed R. Elbestawy, Nehal K. Alm Eldin, Mohamed S. Diab

**Affiliations:** 1 Department of Animal Hygiene and Zoonoses, Faculty of Veterinary Medicine, New Valley University, Kharga, New Valley 1062001 Egypt; 2 Department of Pathology and Clinical Pathology, Faculty of Veterinary Medicine, New Valley University, Kharga, New Valley 1062001 Egypt; 3 Department of Microbiology, Faculty of Veterinary Medicine, New Valley University, Kharga, Egypt; 4 Department of Food Hygiene (Milk Hygiene), Faculty of Veterinary Medicine, New Valley University, Kharga, Egypt; 5 Department of Bird and Rabbit Diseases, Faculty of Veterinary Medicine, Menoufia University, Shebeen El-Kom, 32511 Egypt; 6 Faculty of Veterinary Medicine, Menoufia National University, Menoufia, Egypt

**Keywords:** Campylobacteriosis, Antimicrobial resistance *16S rRNA* gene, Virulence genes, *Zoonosis*, Microbiology, Diseases, Medical research

## Abstract

Campylobacteriosis is a significant zoonosis with major public health implications. This study aimed to investigate the prevalence, molecular characteristics, and antimicrobial resistance of *Campylobacter* spp. in animal (rectal swabs and milk), environmental (fecal, water, and wall swabs), and human samples (stool and hand swabs) in New Valley Governorate, Egypt. Among 573 samples analyzed, *Campylobacter* spp. prevalence was highest in rectal swabs (32.9%) and human stool samples (74.2%), with lower rates in fecal samples (25.9%), milk (25.7%), wall swabs (13.1%), and water (10%). All isolates showed 100% resistance to clindamycin, while being completely sensitive to imipenem and amikacin. Multidrug resistance was found in 90.1% of the isolates, and *16S rRNA* was detected in 90% of randomly selected *Campylobacter* spp. The *hipO, cadF*, and *ceuE* genes were detected in 77.8%, 33.3%, and 22.2% of the isolates, respectively. Phylogenetic analysis of the *16S rRNA* gene showed significant congruence either between the tested isolates and each other or with other isolates in the gene bank, confirming the zoonotic transmission of multi-drug-resistant *Campylobacter* spp. This highlights the urgent need for improved biosecurity on farms, better food handling practices, and heightened public health awareness to mitigate the risk of Campylobacteriosis.

**Supplementary Information:**

The online version contains supplementary material available at 10.1038/s41598-025-13120-1.

## Introduction

Campylobacteriosis, a notable zoonosis, poses significant concerns for public health. The causative agent, *Campylobacter*, is a Gram-negative bacterium found in diverse environments, such as animal feces, water, and soil. This microorganism can inhabit a broad spectrum of animals, including farm animals, poultry, and wild species, which act as potential sources of human infection. Importantly, substandard hygiene practices in animal husbandry, poultry farming, and environmental management play crucial roles in the spread and proliferation of *Campylobacter*. On farms, insufficient biosecurity protocols, contaminated food and water supplies, and unhygienic animal living conditions can contribute to the dissemination of *Campylobacter* within and among animal groups. Furthermore, improper handling of waste and inadequate sanitation in livestock production facilities can result in environmental contamination, thereby increasing the risk of human exposure to the pathogen^1–4^. *Campylobacter* species can be shed in the feces of diseased, recovered, or asymptomatic animals, leading to contamination of the environment, which increases the transmission of infection between herds^5,6^.

Human campylobacteriosis primarily occurs through the consumption of contaminated food and beverages. Poultry and other meat products, particularly when undercooked or improperly managed, are major sources of infection. However, contaminated water, unpasteurized milk, and even fresh products can also transmit *Campylobacter*^1,6,7^. The consequences of *Campylobacter* infection can range from mild gastrointestinal illness to severe complications. While most cases resolve spontaneously, some individuals, such as young children, elderly individuals, and immunocompromised individuals, are at increased risk of developing severe infections, including Guillain–Barré syndrome and reactive arthritis^8,9^.

Polymerase chain reaction (PCR) is a powerful molecular technique for detecting and identifying *Campylobacter* species. *16S rRNA* gene sequencing is a widely used method for species-level identification and differentiation^10^. Furthermore, PCR can be employed to detect the presence of virulence genes, which are crucial determinants of *Campylobacter* pathogenicity. These genes, such as the *hip*O, *ceu*E, and *cad*F genes, encode proteins involved in various virulence mechanisms, including iron acquisition, adhesion to host cells, and modulation of host immune responses. The presence or absence of these genes can provide valuable insights into the potential virulence and severity of *Campylobacter* infections^10–12^.

The emergence of antimicrobial resistance in *Campylobacter* poses a significant threat to public health. The indiscriminate use of antibiotics in both human and animal medicine has contributed to the development of drug-resistant strains, limiting treatment options and increasing the severity of infections^13–15^.

From a one health perspective, this study aimed to isolate *Campylobacter* from humans, animals and the environment. Then studying the sensitivity of isolates from different hosts to antibiotics. Finally, the genetic similarity of three representative isolates was studied to assess the potential of zoonotic transmission and support disease control strategies.

## Methods

### Ethical declaration

This study adhered to the ethical guidelines of the “Institutional Review Board” of New Valley University, Egypt (Approval Number: NVREC 0213-20,249). Informed consent was obtained from all farm owners prior to sample collection, ensuring their understanding of the study objectives and procedures.

### Study area and design

**Fig. 1:**
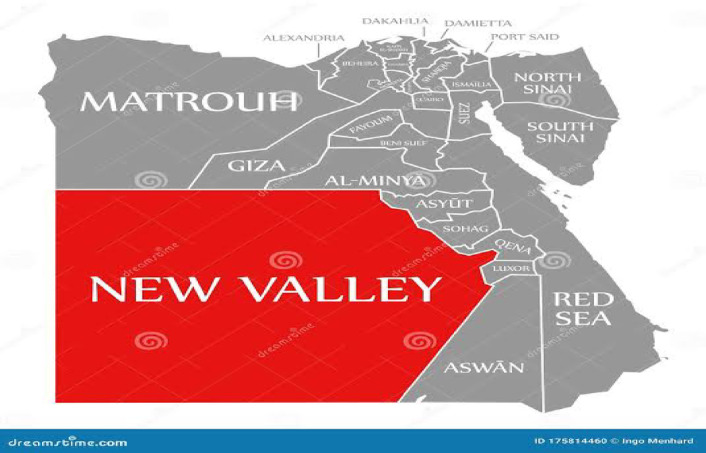
Map of New Valley Governorate showing the locations of the study areas in the Governorate.

The study was conducted in the New Valley Governorate, Egypt, from November 2023 to December 2024. This region encompasses a vast area with diverse agricultural practices, including livestock farming (Fig. 1).

### Sampling

The total number of samples examined is 573 from animals, humans and the environment. Animal samples were firstly, rectal swabs from cows (n = 62), sheep (n = 75) and goats (n = 70). Secondly, the milk samples were from cows (n = 41), sheep (n = 36) and goats (n = 36). As for the human samples, they included: Firstly, 66 stool samples were collected from patients with intestinal disorders who frequent hospitals and laboratories in the study area. Secondly, 46 hand swabs from farm workers and people in contact with animals or their products. As for the environmental samples, they were as follows: 116 fecal samples, 10 water samples from animal courses and 15 wall swabs from animal residences. The samples were withdrawn according to the Campylobacter isolation protocol. All samples were collected under aseptic conditions, labelled and transferred in an ice box to the microbiology lab, Faculty of veterinary medicine New valley university with minimum delay as possible.

### Bacteriological and biochemical analysis of *Campylobacter *spp*.*

*Campylobacter* spp. were isolated according to the ISO 10,272–1 method^16^. Briefly, samples were enriched in Bolton broth (M1592 Himedia, India) supplemented with selective antibiotics and lysed horse blood, then incubated under microaerophilic conditions at 41.5 ± 1 °C for 48 h. Enriched cultures were streaked onto modified charcoal cefoperazone deoxycholate agar (M887I Himedia, India), and incubated under microaerophilic conditions (e.g., gas pack system) at 41.5 °C for 48 h to isolate presumptive *Campylobacter* colonies. Biochemical identification was carried out to the suspected colonies.

### Molecular identification and characterization of *Campylobacter* spp.

PCR was performed to the randomly selected samples to perform molecular confirmation through the detection of the *16S rRNA* gene for species identification and virulence-associated genes (*hip*O, *ceu*E, and *cad*F) via specific primers (Table 1)^17–20^. Genomic DNA was extracted via a QIAamp DNA Mini (catalog no. 51304). Emerald Amp GT PCR master mix (Takara, Code No. RR310A Kit) was used. The PCR products were analyzed via agarose gel electrophoresis and visualized via UV transillumination. The marker for the PCR products was a 100 bp DNA ladder.

**Table 1:** Oligonucleotide primer sequences.

Target gene	Primer sequence (5′-3′)	Length of amplified product	References
*Campylobacter 16S rRNA*	GGATGACACTTTTCGGAGC	816 bp	^17^
	CATTGTAGCACGTGTGTC		
*C. jejuni hipO*	ACTTCTTTATTGCTTGCTGC	323 bp	^18^
	GCCACAACAAGTAAAGAAGC		
*C. coli* ceuE	AATTGAAAATTGCTCCAACTATG	462 bp	^19^
	TGATTTTATTATTTGTAGCAGCG		
*cadF*	TTG AAG GTA ATT TAG ATA TG	400 bp	^20^
	CTA ATA CCT AAA GTT GAA AC		
*Bacterial 16S rRNA*	AGAGTTTGATCMTGGCTCAG	1485 bp	^21^
	TACGGYTACCTTGTTACGACTT		

### Phylogenetic analysis

Phylogenetic analysis was performed on a representative subset of isolates (one animal, one environmental, and one human isolate) on the basis of *16S rRNA* gene sequences (Table 1)^21^. Sequence alignment was performed via CLUSTAL^22^**,** and phylogenetic trees were constructed via MEGA6^23^ via the maximum likelihood, neighbor-joining, and maximum parsimony methods.

### Antibiotic resistance profile of isolated *Campylobacter* spp.

Antimicrobial susceptibility testing was performed via the disc diffusion method. The CLSI (2020) and EUCAST (2021) recommended guidelines breakpoints were used for macrolides, while CLSI breakpoints for Enterobacteriaceae were used for other antibiotics^24,25^. The antimicrobial agents evaluated are listed in Table 2. The multidrug resistance (MDR) index was calculated^26^.

**Table 2:** Antibiotics used and the related breakpoints for interpretation of inhibition zone diameters.

Antimicrobial disc	Concentration (µg)	Sensitive (S) (mm)	Intermediate (I) (mm)	Resistant (R) (mm)
Erythromycin	15	≥ 23	14–22	≤ 13
Ciprofloxacin	5	≥ 21	16–20	≤ 15
Tetracycline	30	≥ 19	15–18	≤ 14
Doxycycline	30	≥ 16	13–15	≤ 12
Gentamicin	10	≥ 15	13–14	≤ 12
Chloramphenicol	30	≥ 18	13–17	≤ 12
Nalidixic acid	30	≥ 19	14–18	≤ 13
Clindamycin	2	≥ 21	15–20	≤ 14
Azithromycin	15	≥ 18	14–17	≤ 13
Amoxicillin-Clavulanic acid	20/10 (30)	≥ 18	14–17	≤ 13
Imipenem	10	≥ 23	16–22	≤ 15
Amikacin	30	≥ 17	–	≤ 17

≤ Equal or less, ≥ equal or more.

### Statistical analysis

Statistical analysis was performed via SPSS, version 25 (IBM Corp., 2013). The data were analyzed via chi-square tests, and the significance level was set at *p* < 0.05. The data were treated as a complete randomization design according to Steel et al^27^.

## Results

### Prevalence

A total of 181 *Campylobacter* spp. isolates were recovered from 573 examined samples. The prevalence of Campylobacter spp. was at Animal samples level 32.9% in rectal swabs and 25.7% in milk samples. Additionally, it was at Human samples level 74.2% in stool samples and 4.4% in hand swabs. Moreover, at Environmental samples level the prevalence was 25.9% in fecal samples from animal litter, 10% in water samples, and 13.3% in wall swabs, as shown in (Table 3).

**Table 3:** Prevalence of *Campylobacter* species in different collected samples.

Source of sample	Type of sample	Species	Total no	Positive		Mean %	Chi-square	*p* value
				No	%			
Animal	Rectal swab	Cattle	62	21	33.87	32.9	0.831	0.362
		Sheep	75	27	36.00			
		Goat	70	20	28.57			
	Milk	Cattle	41	4	9.75	25.7		
		Sheep	36	16	44.44			
		Goat	36	9	25.00			
Environment	Fecal sample	Cattle	52	12	23.07	25.9	8.857	0.012*
		Sheep	31	13	41.93			
		Goat	33	5	15.15			
	Water		10	1	10.00	10.00		
	Wall swab		15	2	13.33	13.3		
Human	Stool		66	49	74.24	74.2	62.821	0.000***
	Hand swab		46	2	4.35	4.4		

Nonsignificant (*p* > 0.05) *: significant (*p* < 0.05) **: highly significant (*p* < 0.01) ***: highly significant (*p* < 0.001).

### Molecular characterization

**Fig. 2:**
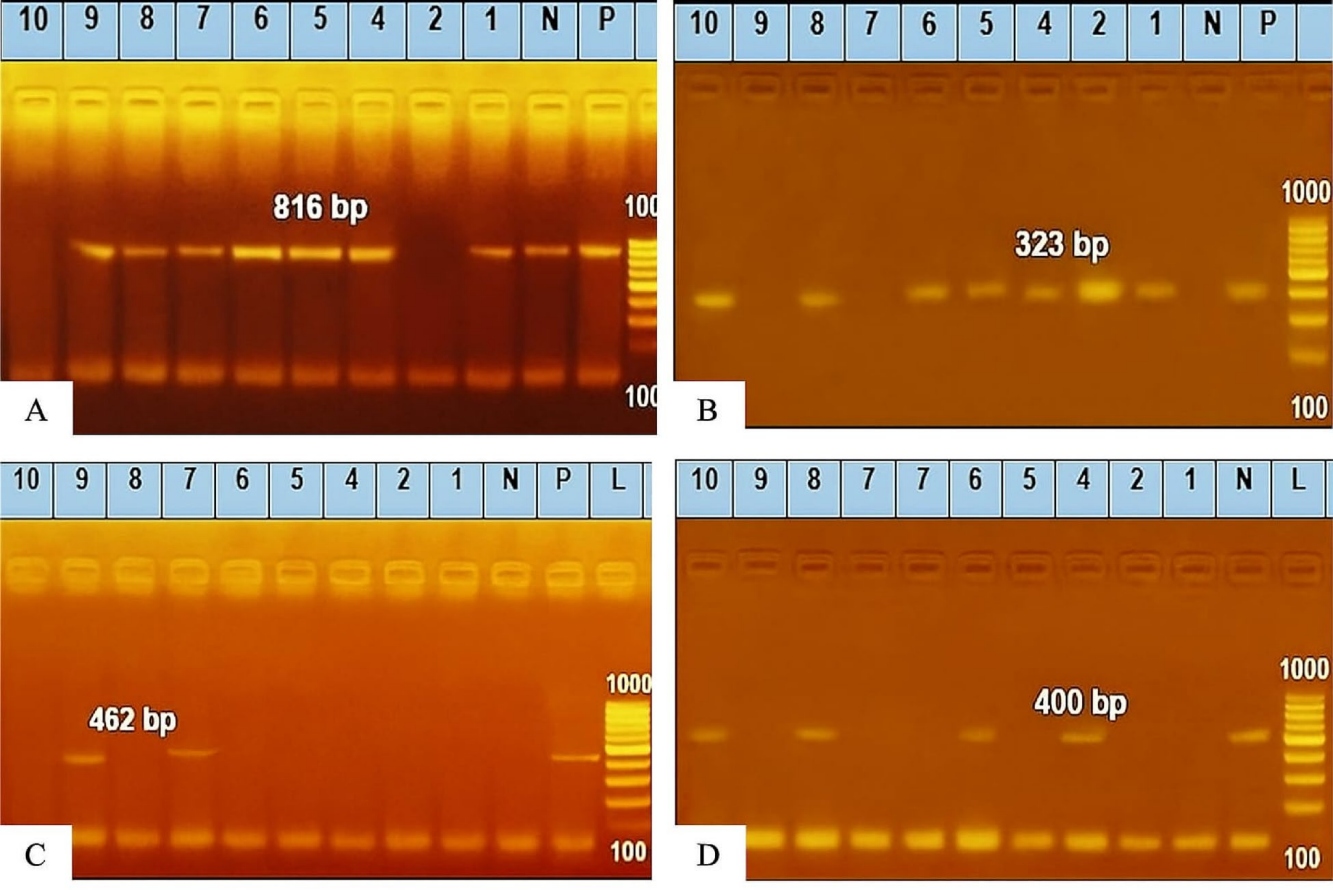
Agarose gel electrophoresis of amplified genes: *16S rRNA* gene with a PCR product of 816 bp) (**A**), *hip*O gene with a PCR product of 323 bp (**B**), *ceu*E gene with a PCR product of 462 bp (**C**) and the *cad*F gene with a PCR product of 400 bp (**D**).

Ten randomly selected isolates were evaluated by PCR for the presence of *16S rRNA* and virulence genes (*hip*O, *ceu*E, and *cad*F). The prevalence of *16S rRNA* was 90% (9/10), whereas the prevalence of virulence genes was 77.8% (7/9) for *hip*O, 22.2% (2/9) for *ceu*E, and 33.3% (3/9) for *cad*F, as shown in Table 4 and Fig. 2.

**Table 4:** Occurrence of virulence genes in 10 randomly selected *Campylobacter* spp. isolates.

Gene	No. of *Campylobacter isolates*	Positive	
		No	%
*Campylobacter 16S rRNA*	10	9	90.00
*C. jejuni hip*O	9	7	77.8
*C. coli ceu*E	9	2	22.2
*Cad*F	9	3	33.3

### Antimicrobial resistance

A total of 181 *Campylobacter* spp. isolates were evaluated for antibiotic susceptibility to 12 commonly used antibiotics in the study area. All the isolates were resistant to clindamycin. In contrast, the highest sensitivity was observed for imipenem and amikacin (100% sensitivity each). The isolates exhibited 96.1% sensitivity to gentamicin, 94.5% sensitivity to doxycycline, and 71.8% sensitivity to ciprofloxacin (Table 5). The mean MAR indices of *Campylobacter* spp. isolated from animal, environment and human samples were 0.393, 0.481, and 0.416, respectively (Table 6). Notably, the highest MAR index was observed in environmental isolates.

**Table 5:** Antibiotic sensitivity of animal, environmental, and human *Campylobacter* spp. isolates.

Antimicrobial agents	Sensitive		Intermediate		Resistant	
	No	%	No	%	No	%
Erythromycin	11	6.08	15	8.3	155	85.6
Ciprofloxacin	130	71.8	29	16.0	22	12.2
Tetracycline	77	42.5	11	6.1	93	51.4
Doxycycline	171	94.5	0	0	10	5.5
Gentamicin	174	96.1	7	3.9	0	0
Chloramphenicol	86	47.5	10	5.5	85	46.96
Nalidixic Acid	95	52.5	22	12.2	64	35.4
Clindamycin	0	0	0	0	181	100
Azithromycin	22	12.1	32	17.7	127	70.2
Amoxicillin-Clavulanic Acid	22	12.2	4	2.2	155	85.6
Imipenem	181	100	0	0	0	0
Amikacin	181	100	0	0	0	0

**Table 6:** Mean MAR indices of the tested *Campylobacter* spp. isolates.

Source of isolates	Mean of MAR index	Mean
Animal	0.393	0.430
Environment	0.481	
Human	0.416	

### Phylogenetic analysis

**Fig. 3:**
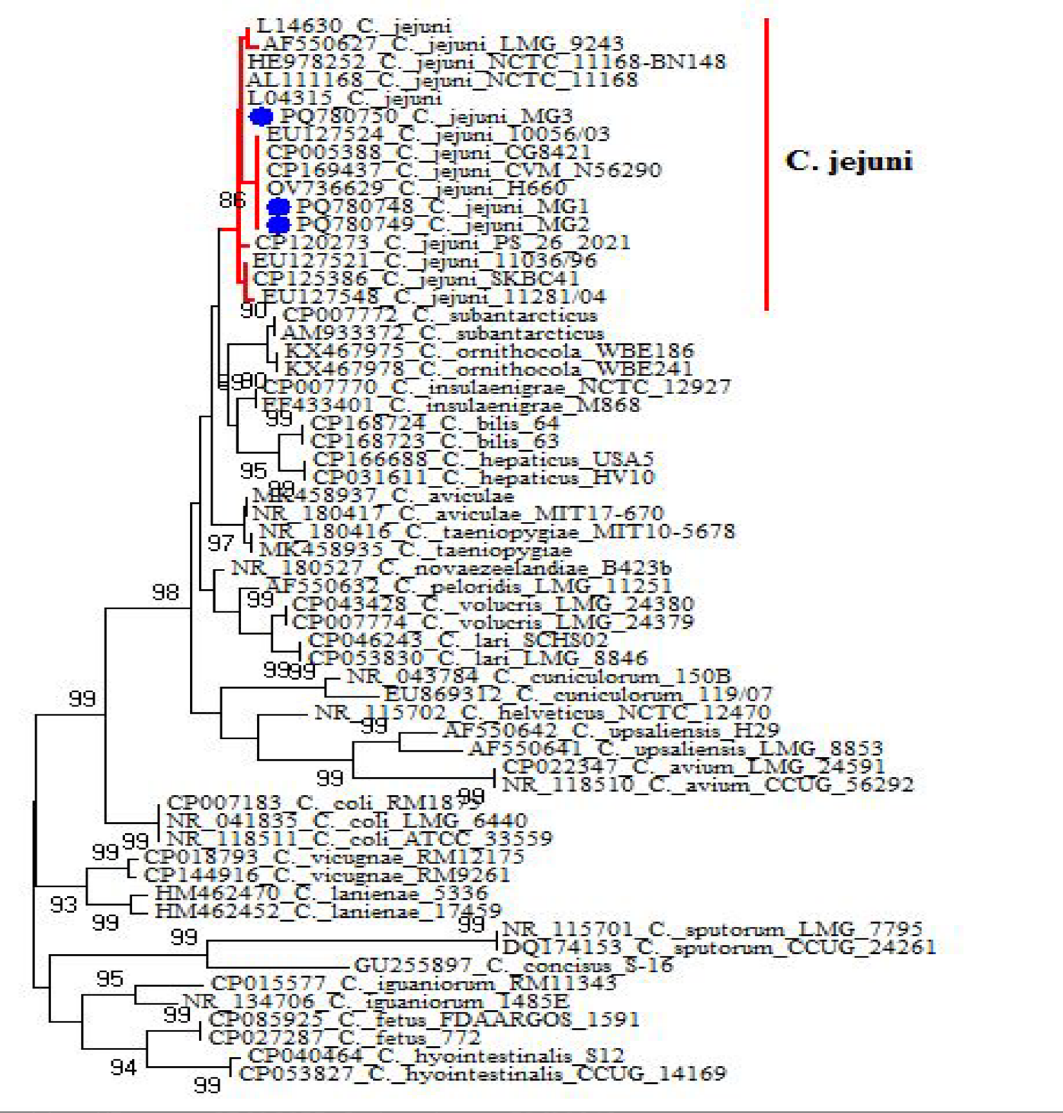
Phylogenetic tree of fecal, milk, and human stool *Campylobacter* spp. isolates based on *16S rRNA* gene.

**Fig. 4:**
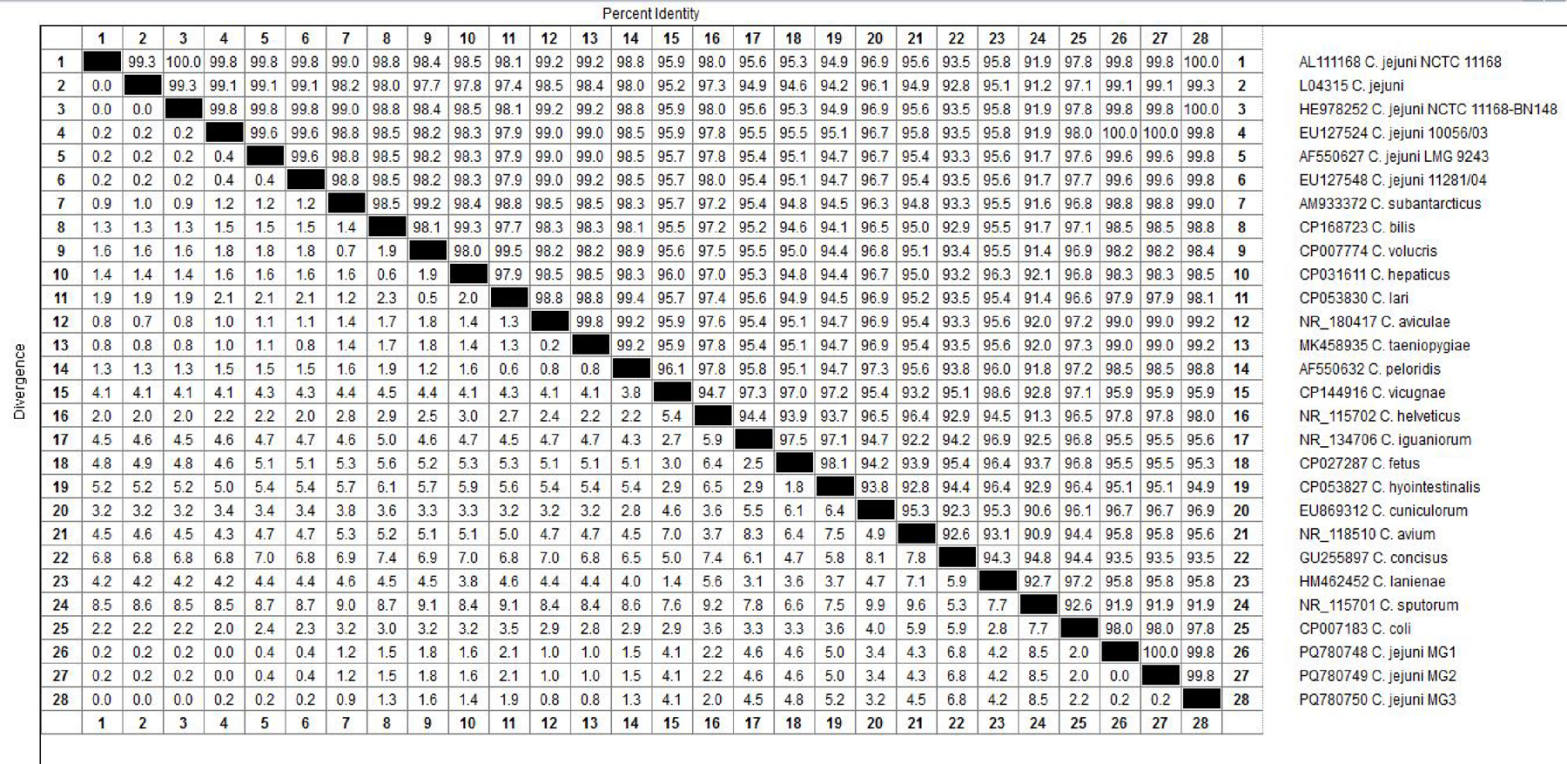
Phylogenetic analysis of animal, human, and environment. *Campylobacter* spp. isolates based on *16S rRNA* gene sequencing.

The *16S rRNA* genes of three randomly selected isolates have been deposited in GenBank under accession numbers: PQ780749 for fecal isolates, PQ780750 for milk isolates, and PQ780748 for human stool isolates (Figs. 3 and 4). Phylogenetic analysis of sequences revealed high genetic similarity between the three isolates ranging from 99.8 to 100%. Additionally, 99.7–100% similarity was found with many other isolates in GenBank.

## Discussion

Campylobacteriosis is a major global public health concern and the leading cause of bacterial gastroenteritis, affecting millions of people annually and contributing to severe complications such as Guillain–Barré syndrome and reactive arthritis. Its burden extends beyond human health, causing infections in livestock, contaminating food and water sources, and imposing significant economic losses on healthcare systems, agriculture, and the food industry^5,28,29^.

The present results revealed that the prevalence of *Campylobacter* spp. in animal rectal swabs was 32.9%. These results were similar to those obtained in Kenya (30.9%)^30^. However, it was higher than the results of previous studies in Assiut, Egypt and Kenya, where the prevalence rates were 20.1% and 15.7%, respectively^31,32^. In contrast, it was lower than the prevalence reported in the United States, Bahama, and Mexico (80.7%)^33^, and 59.2% in northern Spain^34^.

The prevalence of *Campylobacter* spp. in milk was 25.7%. These results were similar to those reported in Italy (24.9%)^35^. However, lower prevalences of 12.6% and 2% were reported in Iraq and Egypt, respectively^36,37^. On the other hand, a higher prevalence rate was reported in Iran (65.8%)^38^.

The prevalence of *Campylobacter* in stool samples was 74.2%, which is similar to findings previously -reported in Egypt (76.9%)^39^. However, it was higher than other results in Egypt and Iran, where the prevalence rates were 66.6% and 8.4%, respectively^6,8^. In contrast, it is lower than the prevalence reported in other Egyptian studies (90.9%)^40^.

The results revealed that the prevalence of *Campylobacter* in hand swabs was 4.4%, which was similar to findings reported among handlers of raw poultry or meat^9^. However, this value was higher than that reported in a previous study in Ethiopia (1.8%)^41^. In contrast, it was lower than the prevalence (11.9%) reported in China^42^, whereas another study could not isolate *Campylobacter* from examined hand swab samples in Egypt^43^. There was a highly significant difference between the human stool and hand swabs (*p* < 0.001).

The prevalence of *Campylobacter* spp. in environmental fecal samples was 25.9%. These results were very close to those of two studies in Canada and Bangladesh (27% and 26.7%, respectively)^29,44^. However, it is lower than the prevalence reported in other studies in Sweden (78%) ^7^.

The prevalence of *Campylobacter* spp. in the water samples was 10%. These findings are similar to those reported in Malaysia (11.1%)^45^; however, these findings are lower than the prevalence reported in another study in South Africa (21.7%)^3^. Notably, no *Campylobacter* was detected in water samples in Bangladesh^5^. Moreover, the prevalence of *Campylobacter* spp. in wall swabs was 13.3%. This finding was similar to the prevalence of 15% reported in Canada^46^, and a previous study reported a prevalence of 15% in poultry farm environments^47^. However, it was lower than the prevalence reported in Malaysia (33.3%)^45^.

PCR is a valuable tool for the molecular confirmation of *Campylobacter* spp., including species identification and the detection of virulence genes^10,11^. In the present study, 90% of the randomly selected isolates were positive for the *16S rRNA* gene. Among the positive isolates, 77.8% (7/9) harbored the *hip*O gene, 22.2% (2/9) harbored the *ceu*E gene, and 33.3% (3/9) harbored the *cad*F gene. These findings are consistent with previous studies reporting the presence of the *hip*O gene in 75% of isolates^48^. However, other studies have reported higher detection rates for the *ceu*E, *hip*O, and *cad*F genes^49,50^. The distribution of genes reflects both species identity and isolation source and the differences between them are always statistically significant. In general, it can be said that The variation in the distribution of these genes may be attributed to factors such as sample sources (e.g., animal. Environment, food and human) *Campylobacter* serotype, geographical location and genetic mutations^12,48,49^.

The high genetic similarity between the three isolates implies that cattle are an important reservoir for human campylobacteriosis. Additionally, these results showed similarities ranging from 99.7 to 100% with many isolates of human and animal origin, such as human stool isolates from the United Kingdom, human stool isolates from a child in Brussels, Belgium, and broiler cloacal swabs from Sweden (Accession Nos. AL111168, HE978252 and AF550627). The phylogenetic relatedness of *C. jejuni* isolates from animals and humans further supports cross-species transmission of this pathogen. These findings are in agreement with those of previous studies that documented zoonotic transmission of *Campylobacter*^51,52^.

The present data revealed that *Campylobacter* isolates presented 100% resistance to clindamycin, followed by high resistance rates to erythromycin, amoxicillin-clavulanic acid (85.64%), and azithromycin (70.2%). These findings are consistent with those of several previous studies^15^. However, they contradict other studies that reported higher susceptibility to these antibiotics^53^. Conversely, the highest sensitivity levels were observed for imipenem, amikacin (100%), gentamicin (96.1%), doxycycline (94.5%), and ciprofloxacin (71.8%). These results align with other studies that reported high sensitivity to imipenem, amikacin, and gentamicin^53^. Our results revealed that 90.1% of the *Campylobacter* spp. isolates exhibited multidrug resistance (MDR), defined as resistance to three or more classes of antibiotics. This finding is consistent with other studies that reported high levels of MDR in *Campylobacter*^32^. In fact, the widespread prevalence of multidrug resistant Campylobacter in the food chain, human, animal or environment has a significant impact on both public health and veterinary in terms of the difficulty of treatment and the increased cost. Consequently, effective strategies to fight antibiotic resistance include responsible antibiotic use and the development of alternative treatments are vital^13,54^.

Our data revealed that the MAR indices of *Campylobacter* spp. isolated from animal, environmental and human samples were 0.393, 0.481, and 0.416, respectively. These findings are consistent with previous studies that reported mean MDR indices of 0.49 for animal isolates^55^, 0.4 for environmental isolates and 0.3–0.4 for human isolates^56^. Notably, the mean MDR index was highest for the environmental isolates, suggesting a relatively high level of antibiotic resistance among *Campylobacter* strains from the environment. Several factors might explain the high antibiotic resistance of environmental isolates, including exposure to antibiotics in the environment through pollution, horizontal gene transfer between bacteria, natural selection due to constant exposure to low levels of antibiotics, and the presence of diverse microbial communities that act as reservoirs for resistance genes^14,57^.

## Conclusions

This study demonstrated a significantly high prevalence of MDR *Campylobacter* spp. in various samples. Phylogenetic analysis revealed a high degree of genetic similarity among isolates from different sources, including animal, environmental, and human samples. This finding underscores the potential for zoonotic transmission of *Campylobacter* spp. between animals, the environment, and humans. Future studies should focus on comparative genomics to identify zoonotic markers and assess the role of agricultural practices in the evolution of *C. jejuni*. Additionally, implementing measures to restrict the use of antibiotics as growth promoters and for prophylaxis on animal farms is crucial to combat antimicrobial resistance. Finally, mitigating the spread of diseases depends critically on a One Health strategy that combines environmental, human, and animal monitoring. Additionally, educating the public on safe food handling practices is essential to prevent human infection.

## Electronic supplementary material

Below is the link to the electronic supplementary material.

Supplementary Material 1

Supplementary Material 2

Supplementary Material 3

Supplementary Material 4

## Data Availability

The datasets generated during and/or analyzed during the current study are provided within the manuscript and supplementary information files.
