# Supplementary material for: Prevalence and molecular characterization of multidrug resistant Campylobacter isolated from animals and humans as a one health approach
Source: Sci Rep. 2025 Aug 18;15:30262. doi: 10.1038/s41598-025-13120-1 (PMC12361403; doi:10.1038/s41598-025-13120-1)
Supplement: Supplementary file 4 — Supplementary Material 4 [file 41598_2025_13120_MOESM4_ESM.pdf]

An official website of the United States government
Here's how you know

Log in

Nucleotide

GenBank

Campylobacter jejuni strain MG3 16S ribosomal RNA gene, partial sequence

GenBank: PQ780750.1

FASTA Graphics

Go to:

```
LOCUS       PQ780750                 960 bp    DNA     linear   BCT 24-DEC-2024
DEFINITION  Campylobacter jejuni strain MG3 16S ribosomal RNA gene, partial
            sequence.
ACCESSION   PQ780750
VERSION     PQ780750.1
KEYWORDS    .
SOURCE      Campylobacter jejuni
  ORGANISM  Campylobacter jejuni
            Bacteria; Pseudomonadati; Campylobacterota; Epsilonproteobacteria;
            Campylobacterales; Campylobacteraceae; Campylobacter.
REFERENCE   1  (bases 1 to 960)
  AUTHORS   Diab,M.S., Sayed,Mg., Kholif,M.E., Alm Eldin,Nk. and Sotohy,S.A.
  TITLE     Prevalence of Campylobacter in human and animal in New Valley
            Governorate
  JOURNAL   Unpublished
REFERENCE   2  (bases 1 to 960)
  AUTHORS   Diab,M.S., Sayed,Mg., Kholif,M.E., Alm Eldin,Nk. and Sotohy,S.A.
  TITLE     Direct Submission
  JOURNAL   Submitted (19-DEC-2024) Department of Animal Hygiene and Zoonoses,
            Faculty of Veterinary Medicine, New Valley University, 1062001,
            kharga Oasis, New Valley 72511, Egypt
COMMENT     Sequences were screened for chimeras by the submitter using chimera
            check 3.

            ##Assembly-Data-START##
            Sequencing Technology :: Sanger dideoxy sequencing
            ##Assembly-Data-END##
FEATURES             Location/Qualifiers
     source          1..960
                     /organism="Campylobacter jejuni"
                     /mol_type="genomic DNA"
                     /strain="MG3"
                     /isolation_source="New-valley"
                     /host="goat"
                     /specimen_voucher="Milk"
                     /db_xref="taxon:197"
                     /geo_loc_name="Egypt: New valley"
                     /collection_date="Oct-2024"
     rRNA            <1..>960
                     /product="16S ribosomal RNA"
ORIGIN
        1 ttcggtgtag gatgagacta tatagtatca gctagttggt aaggtaatgg cttaccaagg
       61 ctatgacgct taactggtct gagaggatga tcagtcacac tggaactgag acacggtcca
      121 gactcctacg ggaggcagca gtagggaata ttgcgcaatg ggggaaaccc tgacgcagca
      181 acgccgcgtg gaggatgaca cttttcggag cgtaaactcc ntttcttagg gaagaattct
      241 gacggtacct aaggaataag caccggctaa ctccgtgcca gcagccgcgg taatacggag
      301 ggtgcaagcg ttactcggaa tcactgggcg taaagggcgc gtaggcggat tatcaagtct
      361 cttgtgaaat ctaatggctt aaccattaaa ctgcttggga aactgatagt ctagagtgag
      421 ggagaggcag atggaattgg tggtgtaggg gtaaaatccg tagatatcac caagaatacc
      481 cattgcgaag gcgatctgct ggaactcaac tgacgctaag gcgcgaaagc gtggggagca
      541 aacaggatta gataccctgg tagtccacgc cctaaacgat gtacactagt tgttggggtg
      601 ctagtcatct cagtaatgca gctaacgcat taagtgtacc gcctggggag tacggtcgca
      661 agattaaaac tcaaaggaat agacggggac ccgcacaagc ggtggagcat gtggtttaat
```

```
      721 tcgaagatac gcgaagaacc ttacctgggc ttgatatcct aagaacctta tagagatatg
      781 agggtgctag cttgctagaa cttagagaca ggtgctgcac ggctgtcgtc agctcgtgtc
      841 gtgagatgtt gggttaagtc ccgcaacgag cgcaacccac gtatttagtt gctaacggtt
      901 cggccgagca ctctaaatag actgccttcg taaggaggag gaaggtgtgg acgacgtcaa
//
```
